# A robust framework for protein-protein interaction prediction with multi-objective ensemble learning and embedding-based representations

**DOI:** 10.1038/s41598-026-55161-0

**Published:** 2026-05-29

**Authors:** Subhashis Chatterjee, Shreya Swarnaker

**Affiliations:** Department of Mathematics and Computing, Indian Institute of Technology (ISM) Dhanbad, Dhanbad, 826007 India

**Keywords:** Computational biology and bioinformatics, Mathematics and computing

## Abstract

Protein–protein interactions (PPIs) perform a key role in virtually all cellular processes. However, experimental identification of PPIs remains costly, time-consuming, and often incomplete. To address these challenges, this study presents a hybrid adaptive framework for PPI prediction that integrates modern protein language models with evolutionary optimization and ensemble learning. It uses the language model Prot-T5-XL-Uniref-50 to embed protein sequences, capturing rich contextual, structural, and physicochemical information. The resulting high-dimensional representations are then compressed using uniform manifold approximation and projection to reduce computational complexity. A hybrid approach coupling the multi-objective non-dominated sorting genetic algorithm-II (NSGA-II) with random forest is then proposed to enhance classifier robustness. This evolutionary strategy simultaneously maximizes prediction accuracy and classifier diversity while estimating the optimal number of trees required for the ensemble from the pareto-optimal fronts. Comparative results with state-of-the-art methods validate the superior performance of the proposed method across four benchmark datasets- *Human*, *E. coli*, *Drosophila*, and *C. elegans*. Finally, using SHapley Additive exPlanations, each feature’s contribution to the model’s predictions was quantified and visualized, facilitating the ranking and examination of influential embedding dimensions. Overall, the proposed framework offers a reliable and robust solution for large-scale PPI prediction based solely on protein sequence data.

## Introduction

In every biological organism, a large portion of cell’s processes and functions are controlled by proteins. Formed by diverse arrangements of twenty standard amino acids, every protein exhibit unique structure and function. Moreover, when a protein is interacting with one or more other proteins, the impact of their collective activity is intensified significantly^1^. Protein protein interactions (PPI) are defined to be physical contacts between two or more proteins through biochemical events. Thorough analysis of PPIs facilitates understanding of various processes, such as metabolism, immunological response, signal transduction, transcription control processes, cellular structure and hormone regulation. Hence, recognizing these interactions are very crucial to comprehend the biological processes that take place within cells, which in turn makes it easier to identify therapeutic targets and create new medications^2–5^. High-efficiency experimental techniques namely, yeast two-hybrid screens (Y2H), Tandem affinity purification (TAP) as well as mass spectrometric protein complex identification (MS-PCI) are capable of yielding large-scale PPI data and proteomic complex composition data; however, they are hindered by significant expense and operational inefficiency due to sluggishness. They also may be incompatible with proteins derived from different kinds of organisms, often resulting in false-positive outcomes. Therefore arised the need of computational techniques to efficiently and accurately predict PPIs. But to develop such a computational model, one would need information of proteins which may be extracted from protein’s sequence, domains or three dimensional structures etc. However, for known proteins, their polypeptide sequence is generally available right away, making it a center of attention for researchers^6–10^.

Literature review shows that there are already some studies present which aim to solve the PPI prediction problem with the help of features extracted from amino acid sequences. The earliest of them used predefined features to this e.g., conjoint triads (CT)^6^, autocovariance (AC)^7^, ontological features^7^, composition-transition-distribution (CTD) descriptors^8^, or coupling more than one among them^9^. Beyond these descriptors, numerous statistical learning techniques have been utilized to infer PPIs^7,10–12^. Another method, SPRINT (Scoring PRotein INTeraction)^13^ infers interaction potential by measuring the impact of recurring sequence patterns across protein sequences . Alternative approaches leverage genomic^14^, proteomic^15^, and structural data^15^. Unfortunately, the scope of interaction information captured by these methods is constrained by the predefined, handcrafted features from protein profiles, which may not fully represent the complexity of PPIs.

To address the shortcoming discussed in previous paragraphs, deep learning algorithms were introduced, enabling the processing of large-scale data and the automatic extraction of task-relevant features. DPPI^16^ utilized a deep convolutional neural network consisted with a Siamese-like design framework, complemented with the help of random projection and data augmentation techniques and is designated to be the first one to employ deep learning for these kind of problem. Wang et al.^17^, and Sun et al.^40^, have employed a stacked auto-encoder to learn the hidden or compact representation of input features, which are derived from protein sequences. HNSPPI^18^ employs a strategy that integrates salient information derived from both protein sequences and network topology, thereby enabling comprehensive feature acquisition. For prediction, it utilizes a simple classifier, maintaining computational efficiency and model lightweightness. Jha et al.^19^ extracted three-dimensional coordinate data from protein structures, subsequently applying ResNet50, autocovariance (AC), and conjoint triads (CT) to derive feature representations. These features were then processed through a long short-term memory (LSTM) network to predict protein–protein interactions. In another study by Jha et al.^20^, a Language Model-based model using a Graph-BERT framework and sequence-derived features were proposed to classify protein-protein interactions. Chen et al.^21^, introduced an ensemble learning approach for PPI prediction that integrated multiple learning algorithms and different protein-pair representations. In a different study by Jha et. al.^22^, efforts were made to employ graph convolutional networks (GCNs) and graph attention networks (GATs) for predicting protein-protein interactions by leveraging both structural information and sequence-derived features of proteins. AutoPPI^23^, employs ensemble of autoencoders which consist of three type of neural network architechtures and represents protein sequences by a combination of conjoint triad method and autocovariance to perform the binary classifiaction. Yang et al.^24^, utilized an improved graph representation learning method which can extract information from both sequence and graph structure of proteins and shows superiority over many existing models.

Although deep learning has shown strong performance in addressing the problem in consideration, it is crucial to recognize that deploying such models demands significant memory, storage, computational complexity, and considerable time investment. Moreover, different datasets are collected and curated using diverse methodologies, ensuring variety among them; therefore, it is unrealistic to expect the same model with identical hyperparameters to perform effectively across all datasets.

This created the need for a model capable of handling diverse data samples while assessing the significance of specific attributes for prediction using less storage space and lesser computational time. The motto is to produce a model that is adaptive; the framework is expected to dynamically optimize its own parameters to match the unique characteristics of different PPI datasets.

**Fig. 1:**
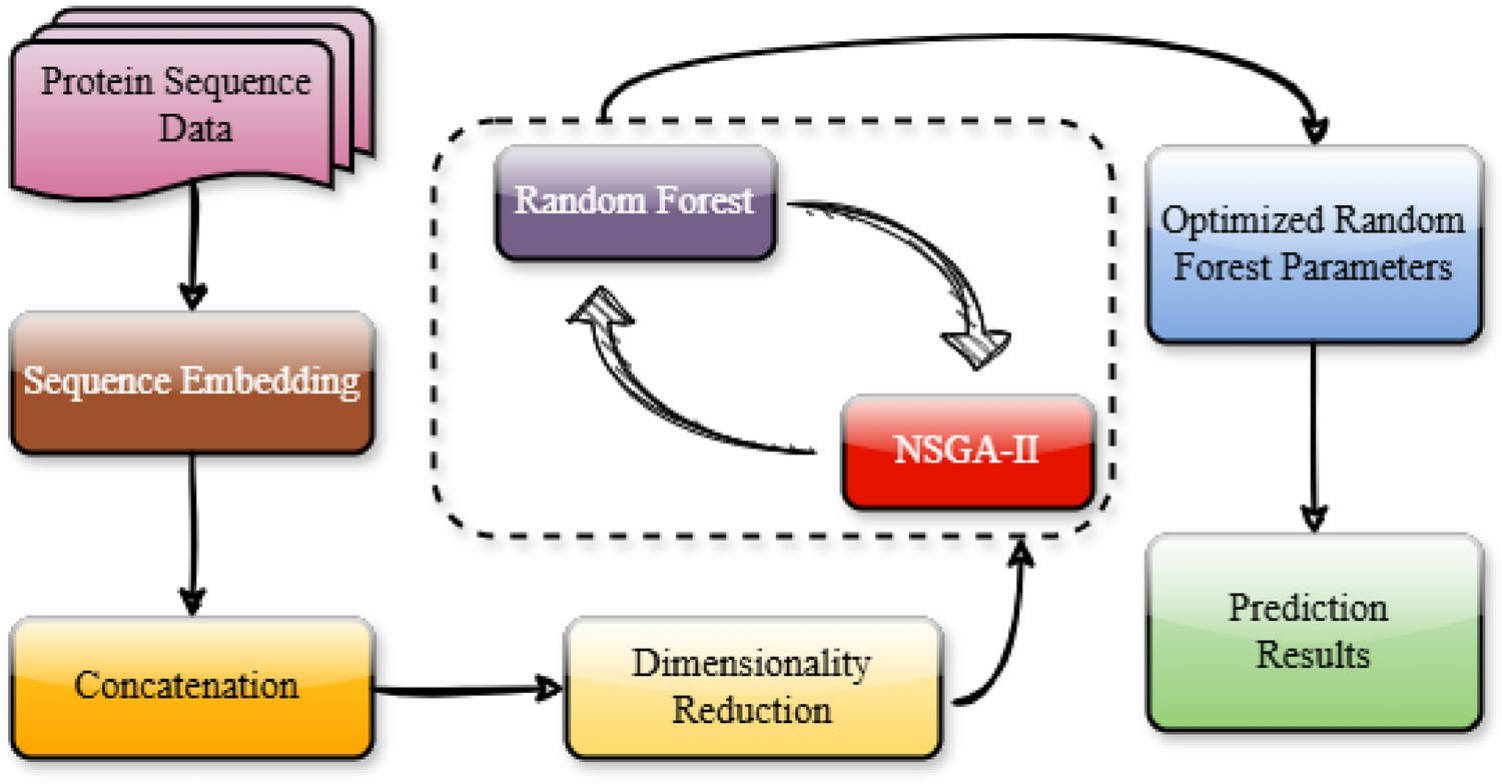
Architecture of the proposed hybrid NSGAII-RF model. This figure represents the basic architecture pipeline of the proposed model.

This paper proposes a distinctive approach to achieve the aforementioned goal. Initially, the protein sequences are embedded using a protein language model, Prott5-XL-Uniref50^25^, which is able to capture multifaceted nature of individual amino acids in a protein, contextual relationships, physicochemical characteristics, evolutionary conservation, and functional importance. To reduce computational complexity and improve processing efficiency, the representations of protein sequences are projected into a lower dimensional space using uniform manifold approximation projection (UMAP)^37^, a highly scalable and computationally efficient way, capable of handling large datasets. Here the default transductive manifold learning formulation has been adopted, following the principle that when the full set of instances is known in advance, jointly optimizing the embedding over all samples produces a geometrically superior representation- which aligns with this study’s objective of predicting interactions within a pre-defined interactome. A hybrid computational framework is proposed for PPI prediction that integrates the non-dominated sorting genetic algorithm (NSGA)-II with the random forest classifier. The primary objective of this approach is to simultaneously determine the near-optimal number of decision trees required for the ensemble while ensuring that the generated trees exhibit both high individual accuracy and significant diversity. To achieve this, the conventional mechanisms of Random Forest (RF) algorithm, specifically, bootstrap aggregating (bagging) and random feature selection are substituted with NSGA-II algorithm. This approach optimizes the composition of the training sets instead of relying on random selection. The required hyperparameters for constructing the Random Forest are determined by selecting solutions from the Pareto-optimal front. As a result, each decision tree is trained on a distinct dataset defined by a unique combination of samples and feature subsets. This evolutionary strategy enables the construction of a robust ensemble in which the trade-off between classification accuracy and diversity is more effectively controlled than in conventional methods. The proposed hybrid NSGA-II-RF model is then trained and evaluated on four widely used benchmark datasets—*Human*, *Escherichia coli*, *Drosophila* melanogaster, and *Caenorhabditis elegans*. The results demonstrate consistent improvements over existing state-of-the-art approaches, highlighting the efficiency and reliability of the proposed classification framework. Figure 1 illustrates the architecture of the proposed hybrid NSGA-II–RF model.

The remaining parts of the paper are organized as follows: Section "Methodology" describes the methodology in detail covering architecture and training process, along with the accuracy metrics employed. Section "Results" outlines the dataset, obtained results, coverage analysis and explainability of the model. Section "Significance and complexity" discusses the statistical significance and computational complexity of the methods. Section "Comparisons" presents comparison of the proposed model with state-of-the-art models in terms of accuracies. Section "Discussion" provides an in-depth discussion of the results achieved throughout the study. Finally, Section "Conclusion" offers concluding remarks.

## Methodology

The main goal in this paper is to improve the performance of the random forest algorithm through creating diverse and well-performing training sets in order to generate accurate and diverse classifiers. Since there is a trade-off between diversity and accuracy^28^, these two criterias need to be simultaneously optimized to generate accurate and diverse decision trees and determine the near-optimal number of trees.

Determining the optimal configuration for quantizing a random forest model can be framed as a multi-objective optimization problem. This involves identifying a configuration that simultaneously minimizes classification error and maximizes diversity of the classifiers. In ensemble models, diversity refers to the variations among individual classifiers. To achieve an improvement in a solution by combining different classifiers, these classifiers must exhibit differences. Hence, enhancing the performance of the ensemble relies on the diversification of its base classifiers, as long as this does not compromise with the accuracy of the individual components. Multi-objective optimization algorithms such as NSGA-II are well suited for identifying effective model configurations. In this work, NSGA-II is used in combination with ProtT5-XL-UniRef50 embeddings to optimize the Random Forest configuration.

The general architecture of the prospective model is as follows. It encompasses five distinct phases: encoding amino acids, learning protein pairs , dimensionality reduction, intermediate optimization phase, and prediction. Each phase plays a crucial role in the overall architecture and functionality of the model.

- During encoding, protein sequences are converted into vector representations using the ProtT5-XL-UniRef50 protein language model. These embeddings capture rich information from the sequences, including evolutionary signals as well as underlying physicochemical and structural characteristics.
- The second phase captures the mutual information between protein pairs by concatenating their respective embeddings. This step facilitates the construction of a unified representation that encapsulates the interactive dynamics inherent to each protein pair.
- The third phase reduces the dimensionality of the representation of the protein pairs by employing the nonparametric graph-based dimensionality reduction algorithm UMAP.
- The fourth phase serves as the principal stage, in which the training data is passed through the hybrid NSGAII-RF model to tune the hyperparameters of RF algorithm.
- The prediction phase leverages the model with proper set of hyperparameters and trained on the refined depiction of protein sequence pairs to amplify the model performance.

**Fig. 2:**
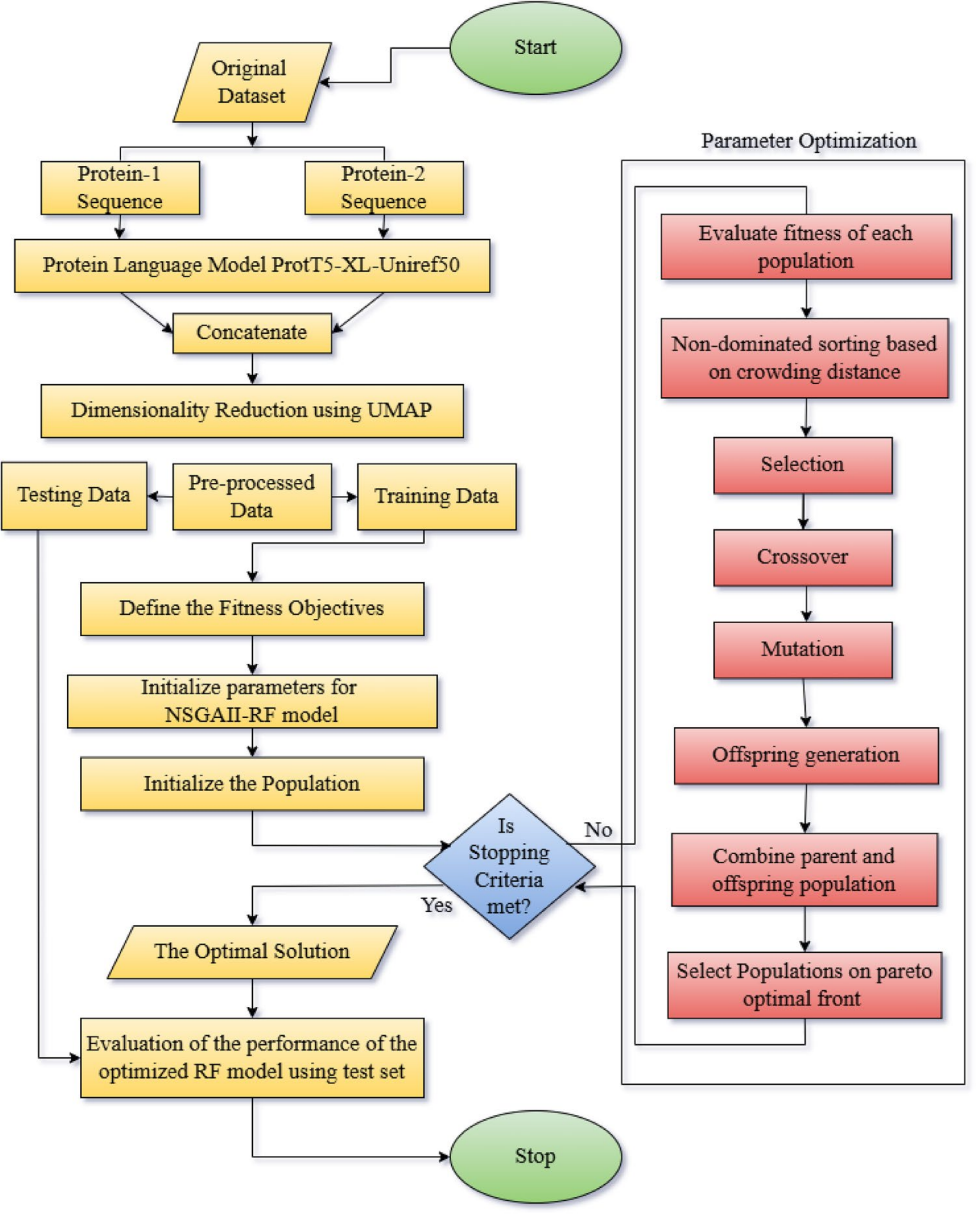
Flowchart of the proposed hybrid NSGAII-RF model. This figure provides a clean and detailed flowchart for the proposed method including each key steps.

Figure  2 provides the detailed workflow of the proposed technique.

### Embedding protein sequence

The language model ProtT5-XL-UniRef50 is adapted from the T5 architecture, designed to process protein sequences effectively. The T5 Transformer architecture, employs an encoder-decoder structure and includes 3 billion parameters. The model was initially trained on the BFD dataset, which contains over 2,000 million proteins and then fine-tuned on UniRef50, a curated and less redundant dataset with 45 million sequences. This language model tokenizes Protein sequences at the amino acid level, where each residue is treated as a token. By harnessing the power of the protein language model prott5-xl-uniref50^25^, the proposed model achieve a competent understanding of the complex variances inherent in biological sequences, leading to the generation of more accurate and informative protein representations.

To signify the interaction between two proteins, their respective embeddings are concatenated. The same is done if two proteins are known to be not interacting. Therefore every protein pair in question can be depicted as a 1 *×* 2048 vector hereafter. This representation makes a way for protein pairs to bring their respective information into a shared feature space and thus providing a opportunity to capture interaction-specific patterns and dependencies.

### UMAP

UMAP^37^ is a completely unsupervised, nonparametric graph-based dimensionality reduction algorithm using applied Riemannian geometry and algebraic topology to find low-dimensional embeddings of structured data. The UMAP algorithm consists of two steps: (1) computing a graphical representation of a data set (fuzzy simplicial complex) and (2) through stochastic gradient descent, optimizing a low-dimensional embedding of the graph. This optimization process ensures that the local and global structures of the data are preserved as accurately as possible. This approach is by default a transductive approach and works on the given dataset as a whole. UMAP is highly scalable and computationally efficient, capable of handling large datasets with ease and well-suited for visualizing and clustering data in lower dimensions.

In the proposed study, the UMAP algorithm was fit on the global feature distribution of each target proteome, prior to train test split. No access to any interaction label was provided at any stage. Thereafter, the classification model was trained on the training labels and tested on the held-out testing labels, ensuring a strict blind label protocol throughout. This transductive dimension reduction stategy was deliberately chosen, since the aim in this study is to screen interaction within a fixed, pre-defined proteome and not an universal generaliztion. It optimizes the low-dimensional co-ordinate system for the exact protein manifold under study, maximizing geometric faithfulness.

**Fig. 3:**
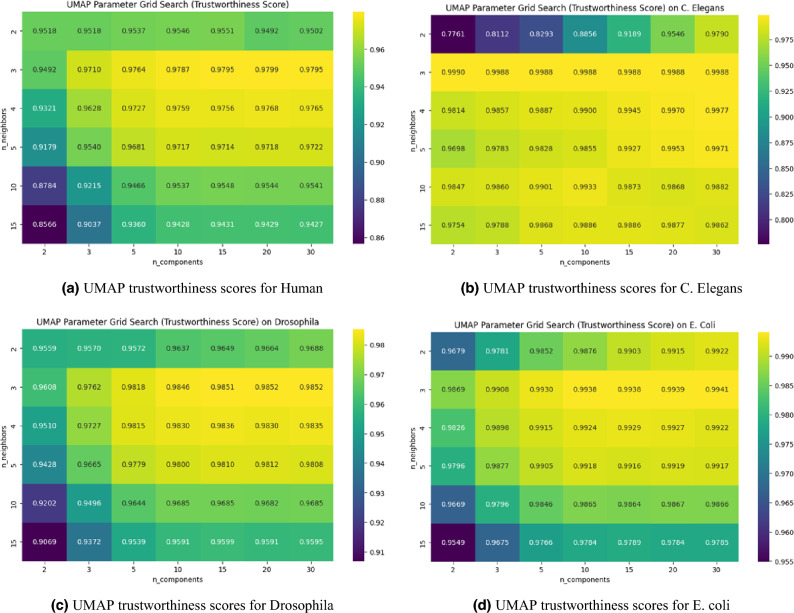
UMAP parameter grid search results showing the trustworthiness score for different organisms across different combinations of n_neighbors and n_components. Lighter colors indicate higher scores i.e., better preservation of local structure.

The output embeddings from the protein language model (PLM) were given as input to UMAP for dimension reduction. To determine the optimal dimensionality for the embedding, a systematic parameter grid search across n_components *∈* {2,3,5,10,15,20,30} and n_neighbors *∈* {2,3,4,5,10,15} has been done to check the manifold trustworthiness, according to^42^. The resulting trustworthiness score heatmaps are shown in Fig. 3.

Following the heatmaps, it is very clear that there are very minimal gains in trustworthiness score if the n_components is increased beyond 3. At n_components=3, the scores are 0.9710 (*Human*), 0.9988 (*C. elegans*), 0.9762 (*Drosophila*), and 0.9908 (*E. coli*), all exceeding 97%. However, moving from n_components=3 to n_components = 30 yields a score improvement of around 1% across all datasets, proving that a dimension of 3 is capable to capture meaningful geometrical structures in the manifold. Therefore, the embedding dimension *d=3* was further used in the downstream classifier.

### Random forest algorithm

The supervised random forest^26^ algorithm operates based on the fundamental principle of bootstrap aggregation, commonly referred to as bagging, applied to the decision tree (DT) algorithm. Given a training dataset $X=\{x_1, x_2,..., x_n\}$ with corresponding outputs $Y=\{y_1, y_2,..., y_n\}$, bagging involves repeatedly selecting random samples, with replacement, from the training set over N multiple iterations. Decision trees are then constructed and fitted to these sampled subsets of the data, enabling the model to reduce variance and improve robustness. Further, all the individual trees are employed to generate predictions for the test samples. The final prediction is then derived by averaging the outputs from all the individual trees, a process that leverages the bagging technique to enhance prediction accuracy and model stability. Mathematically, $f=\frac{1}{N}\sum _{n=1}^{N}f_n(x)$, where, $f_n(x)$ is the $n^{th}$ tree trained on the $n^{th}$ batch of random samples and *x* is the testing sample.

The bootstrapping procedure enhances the model’s overall performance by reducing variance without increasing bias. While individual decision trees are prone to overfitting, averaging the predictions of multiple trees mitigates the impact of noise. This approach makes the model highly robust, as the randomness introduced in selecting both the samples and the features for each tree effectively minimizes correlations among the trees. However, further improvements in the model’s classification accuracy are necessary to enhance its predictive capability of interactions between proteins.

The crucial parameters of RF that are to be optimized in this study are n_estimators, max_depth, min_sample_split and min_sample_leaf where n_estimators is the required number of decision trees in the RF model, max_depth refers to the maximum depth of the generated decision tree, min_sample_split specifies the minimum number of samples required to split an internal node, while min_samples_leaf specifies the minimum number of samples required to be at a leaf node.

### NSGA-II algorithm

NSGA-II is a genetic algorithm designed for multi-objective optimization^27^. It is distinguished by three key features: (1) it employs an elitist approach, allowing the best-performing individuals in a population to progress to the next generation, (2) it incorporates crowding distance to explicitly preserve diversity within the population, and (3) it prioritizes non-dominated solutions.

NSGA-II employs non-dominated sorting for organizing individuals into ranks based on dominance. Individuals not dominated by any other are assigned to front number 1, also called Pareto optimal front. Those dominated only by individuals in this front are assigned to front number 2, and the process continues similarly for subsequent fronts. Solutions on the Pareto front represent a balance between different objectives and represent the set of best-in-class solutions that the algorithm discovers. Selection is carried out via a tournament between two individuals. If the individuals belong to different fronts, the one with the lower front number is selected. If they are within the same front, the individual with the higher crowding distance is chosen, as this indicates a position in a less densely populated area of the front, promoting diversity. In each iteration, N new individuals (offspring) are generated. The offsprings are combined with the original parents to make 2N population which again competes in next iteration. This competition ensures that the next generation consists of the best-performing individuals, maintaining a balance between exploration and exploitation. The iterations stop when it reaches to a certain predefined value.

In the present work, the algorithm employed a population size of 200 individuals, with 100 offspring generated per generation. The initial population was created using random continuous sampling, ensuring a diverse exploration of the search space at the start of the optimization process. For genetic variation, Simulated Binary Crossover (SBX) was applied with a crossover probability of 0.9 and a distribution index (*η*) of 15. This operator promotes the generation of offspring near parent solutions while still allowing sufficient exploration, making it suitable for real-valued decision variables. Mutation was performed using polynomial mutation (PM) with a distribution index (*η*) of 20, enabling controlled perturbations of decision variables to avoid premature convergence and enhance local search capabilities.

**Fig. 4:**
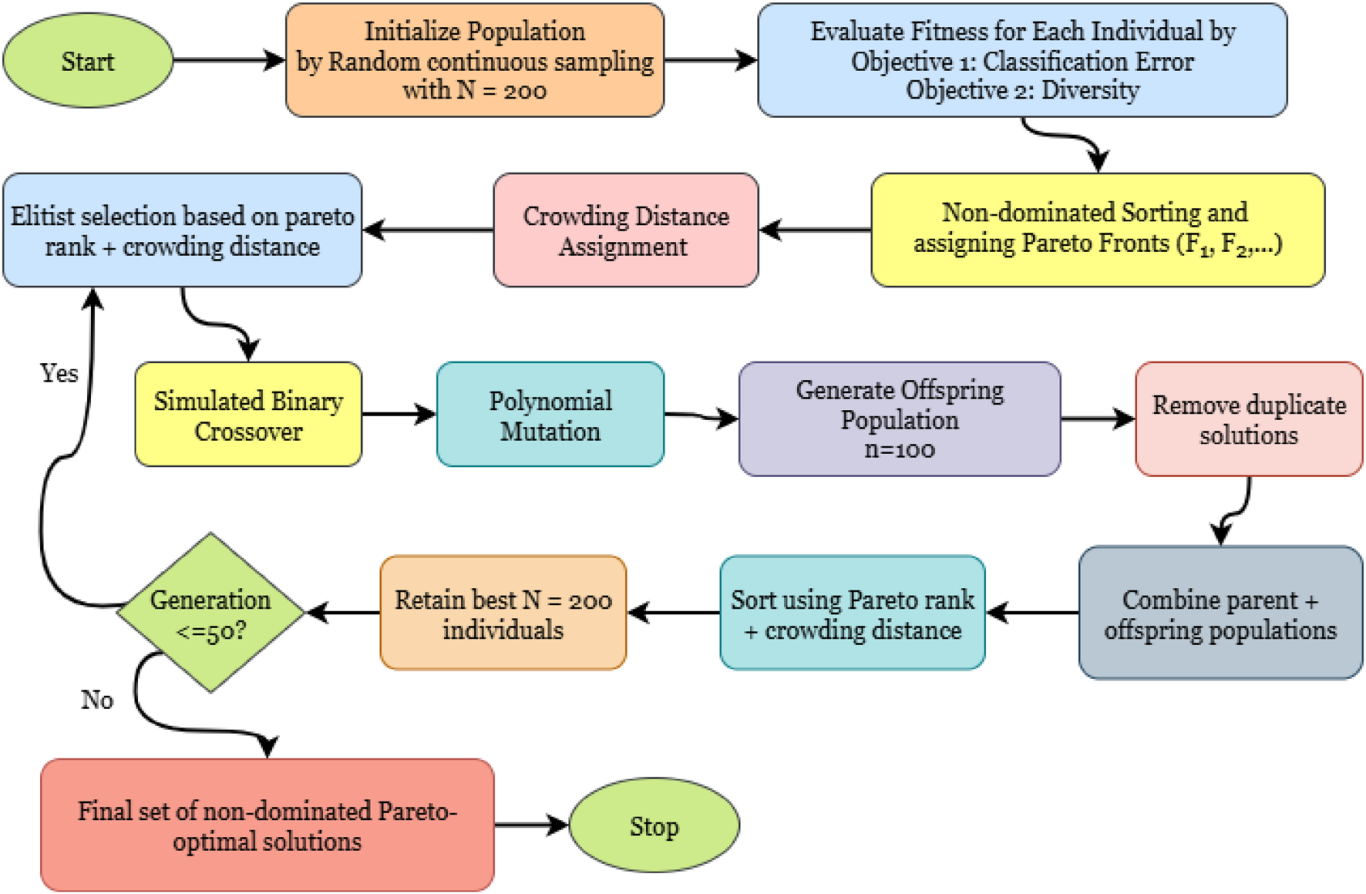
This figure shows the workflow of the bi-objective NSGA-II algorithm with all technical details and adptations used in this study.

To preserve population diversity, duplicate solutions were eliminated during the evolutionary process. Selection was based on non-dominated sorting, where solutions were ranked according to Pareto dominance, and crowding distance, which ensured diversity along the Pareto front by favoring solutions in less crowded regions. The algorithm was run for 50 generations with a fixed random seed for reproducibility. Workflow of this algorithm is depicted in Fig. 4

Each candidate solution produced by the NSGA-II algorithm represents a vector of random forest hyperparameters:

1 $$\begin{aligned} x = (n_{estimators}, max\_depth, min\_sample\_split, min\_sample\_leaf) \end{aligned}$$

For each such candidate solution, a random forest classifier is instantiated using the corresponding hyperparameter configuration and trained on the training dataset.

### Fitness function

The fitness function employed to evaluate candidate solutions is formulated as a bi-objective optimization problem, simultaneously considering classification performance and model diversity. Let *s* denote a candidate solution (i.e., a Random Forest hyperparameter configuration)^28^. Then the optimization objective can be defined mathematically as $minimize (f(s))= min(f_1(s), f_2(s))$ where $f_1(s)$ denotes classification error and $f_2(s)$ denotes diversity-related objective.

*Objective 1: classification error*

The first objective function $f_1(s)$ measures predictive performance and is defined by

$$\begin{aligned} f_1(s)= classification\_error= \frac{FP+FN}{TP+TN+FN+FP} \end{aligned}$$

where *TP*, *TN*, *FP* and *FN* respectively denote true positives, true negatives, false positives, and false negatives. Since the optimization framework follows a minimization paradigm, classification accuracy is equivalently optimized by minimizing the classification error.

*Objective 2: diversity measure*

The second objective function $f_2(s)$ is utilized to quantify the diversity of potential solutions and control the structural complexity of the model. To enhance diversity in ensemble approaches is by utilizing various training subsets and different feature subsets. However, unlike traditional ensemble diversity measures that rely solely on data resampling or feature subspacing^28^, an augmented version of the diversity formulation that integrates data characteristics and ensemble structure has been introduced. Specifically, diversity is defined as a composite measure consisting of three components: data compactness, tree count, and cardinality.

$$\begin{aligned} f_2(s) = diversity = data\_compactness+ \frac{1}{|\tau |}+ \bar{d} \end{aligned}$$

where *|τ |* represents the number of trees in the Random Forest ensemble and $\bar{d}$ denotes the average depth of the trees.

*Data compactness:* The data compactness criterion evaluates the spread of the training set. Unlike the Euclidean distance, the Mahalanobis distance is used here, as it accounts for both scale independence, and covariance, making it robust against noise and redundant data in the training set. The Mahalanobis distance $D^2$ is calculated for each data point relative to the mean using the covariance matrix for a dataset $G_{n\times m}$. The compactness is then computed as the inverse of the average distance as,

$$\begin{aligned} Compactness=\frac{1}{ \frac{1}{n}\sum _{i=1}^n {D_i}^2} \end{aligned}$$

The compactness value reflects the degree of scatter in the dataset; a lower value indicates higher dispersion and, consequently, greater diversity among data samples.

*Tree count:* The decision trees in a random forest model are trained on different subsets of data, capturing varied patterns and reducing overfitting. While increased number of trees can improve performance, they also increase computational complexity. The ’tree count’ criterion seeks to minimize the number of trees needed to construct the random forest model, thereby reducing computational complexity and training time.

*Cardinality:* Cardinality captures the structural complexity of the ensemble by measuring the average depth of the decision trees i.e., it indicates to the count of features selected here.

These three components simultaneously reduces classification and ensures that the complexity of the overall model is maintained. This approach allows for the creation of an efficient model with favorable trade-off between predictive accuracy, ensemble diversity, and computational efficiency.

The Algorithm 1 illustrates the steps involved in the advised multi-step hybrid prediction technique.

**Algorithm 1:**
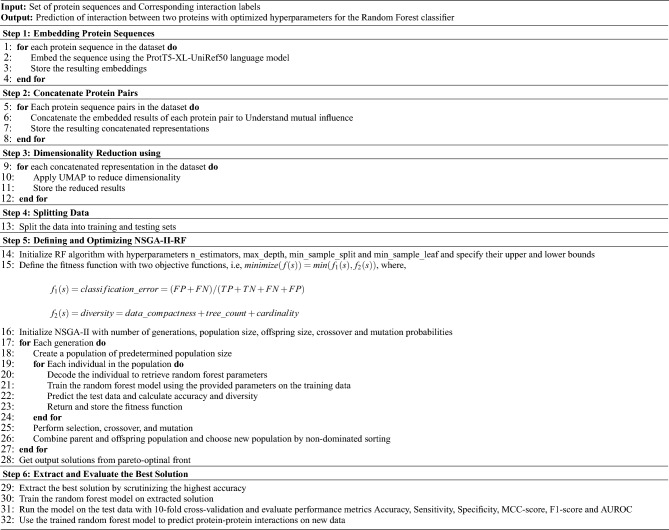
Proposed multi-step hybrid PPI prediction Algorithm

## Results

### Dataset

This study adopts the human PPI dataset as a benchmark, accessible via http://www.csbio.sjtu.edu.cn/bioinf/LR_PPI/Data.htm, commonly referred to as Pan’s *Human* dataset^29^. The positive interaction pairs were curated from the 2007 edition of the Human Protein Reference Database (HPRD), comprising protein couples known to interact. Negative pairs were generated by randomly combining proteins with distinct subcellular localizations, following the procedure outlined by Pan et al.^29^, with additional non-interacting examples sourced from the Negatome database^30^. The final dataset includes 36,630 positive PPI pairs involving 9,476 unique proteins, and 36,480 negative pairs derived from 2,184 proteins.

Datasets from three other species, *E. coli*, *Drosophila* and *C. elegans*, provided by Guo et al.^31^ are also used to examine the predictive capability of the proposed framework. The *E. coli* dataset contains 1528 proteins with 5576 positive samples and 4031 negative samples. The *Drosophila* dataset contains 7059 proteins with 19712 positive samples and 14900 negative samples. Lastly, the *C. elegans* dataset contains 1734 proteins with 2877 positive samples and 1670 negative samples. To create non-redundant PPI subsets of these species, the CD-HIT program^32^ has been used. Proteins exhibiting sequence identity greater than 40% or comprising fewer than 50 amino acids are excluded from the dataset.

To further confirm data integrity, symmetric duplicate pairs, where both (A, B) and (B, A) appeared as separate entries, were identified and removed. Following this and stratified splitting, it was confirmed that zero interaction pairs appear in both the training and test sets, and zero pairs are contradictorily labelled across the positive and negative sets. The class distribution was also checked and found to be perfectly balanced and consistent across the train-test boundary, ruling out class imbalance as a contributing factor to accuracy.

### Result

The NSGA-II search produced a set of Pareto-optimal solutions balancing predictive accuracy, classifier diversity, and model complexity. For each species-specific dataset, the final model was selected from the Pareto front using the Fitness Evaluation score. Table 1 summarizes the optimized parameters obtained after convergence.

**Table 1:** Optimized parameters for all the datasets.

Dataset	n_estimators	max_depth	min_samples_split	min_samples_leaf
*Human*	188	15	2	1
*E. coli*	10	8	5	1
*Drosophila*	339	486	2	1
*C. elegans*	14	6	7	1

This table shows the hyper parameters for each datasets after they have been optimized using the proposed methodlogy

After selecting the optimal hyperparameters, the optimized model was evaluated using the 10-fold cross-validation framework. To assess the performance, several accuracy metrics, such as: accuracy, sensitivity, specificity, F1-score, Matthews correlation coefficient (MCC) and area under the receiver operating characteristic (AUROC) curve are used.

$$\begin{aligned} accuracy = \frac{TN+TP}{TN+TP+FN+FP} \end{aligned}$$

$$\begin{aligned} Sensitivity = \frac{TP}{TP+FN} \end{aligned}$$

$$\begin{aligned} Specificity = \frac{TN}{TN+FP} \end{aligned}$$

$$\begin{aligned} f1-score=\frac{TP}{TP+\frac{FP+FN}{2}} \end{aligned}$$

$$\begin{aligned} MCC=\frac{TP\times TN-FP\times FN}{\sqrt{(TP+FP)(TP+FN)(TN+FP)(TN+FN)}} \end{aligned}$$

**Table 2:** Performance of proposed model on the datasets.

Dataset	Acc.(%)	Sens.(%)	Spec.(%)	MCC(%)	f1(%)	AUROC(%)
*Human*	99.81 ± 0.04	99.86 ± 0.03	99.90 ± 0.07	99.32 ± 0.09	99.92 ± 0.01	99.90 ± 0.00
*E. coli*	99.99 ± 0.01	99.98 ± 0.00	1.00 ± 0.00	99.97 ± 0.00	99.99 ± 0.00	1.00 ± 0.00
*Drosophila*	99.32 ± 0.20	99.79 ± .01	99.77 ± 0.01	98.83 ± 0.01	99.97 ± 0.00	99.98 ± 0.01
*C. elegans*	99.99 ± 0.01	1.00 ± 0.00	99.94 ± 0.04	99.95 ± 0.04	99.98 ± 0.00	1.00 ± 0.00

Shows the different metric values for each data after the prediction has been done on the test data with optimized Random Forest models

Till date, numerous studies have been conducted to predict PPIs using artificial intelligence-based approaches. Following the state-of-the-art methods, in this study, the whole dataset (positive and negative data merged together) is divided into a training set (80%) to train the model and a test set (20%) to evaluate the model’s performance. The optimized model demonstrates consistently strong performance across all four species, reaching near-perfect discrimination in several cases. It is shown that for the Pan’s *Human* dataset, a maximum of 99.41% accuracy has been achieved, for the *Drosophila* dataset, a maximum of 99.42% accuracy has been achieved, while for both *E. coli* and *C. elegans*, 100% maximum accuracies are obtained. Table 2 depicts the evaluation metrics for each dataset.

Additionally, to prove that the classifier learns meaningful interaction determinants rather than remembering, two more strong evaluations has been added: (a) a C3 style evaluation and (b) Hard negative experiment.

Under C3 style, neither protein in a test pair is encountered during training, representing a qualitatively harder prediction problem than C1, where the model might have encountered both proteins of a test pair during training. The methodology for this kind of evaluation was established by^38^. The corresponding results have been provided below in the Table 3 alongside the C1 (where data is randomly split into training and testing set) results.

**Table 3:** Results of C3 style evaluation over all datasets and comparison with C1 (random splitting) style evaluation.

Datasets	C1 accuracy (%)	C1 AUROC (%)	C3 Accuracy (%)	C3 AUROC (%)
*Human*	99.81 ± 0.04	99.99 ± 00	85.16 ± 0.30	86.57 ± 0.02
*E. coli*	99.99 ± 0.01	1.00 ± 00	91.08 ± 0.10	87.23 ± 0.26
*Drosophila*	99.32 ± 0.20	99.98 ± 0.01	80.04 ± 0.20	76.72 ± 0.41
*C. elegans*	99.99 ± 0.01	1.00 ± 00	90.47 ± 0.15	81.37 ± 0.25

This table shows the evaluation results when done under C3 (no protein from test data is seen in training data) style. Also, compares it with C1 style (random solitting)

To our best knowledge, this result is the first C3 evaluation report on Pan *Human* data and Guo multispecies data, therefore no direct comparison being readily available, and establishing a new reference point for future comparisons.^38^ showed that the performance accuracy frequently drops by 20-40% and in some cases to near-random level. The performance reduction from C1 to C3 is therefore consistent with the universal pattern documented in literature and is and mathematically expected consequence of the increased difficulty.

In the hard negative test, same-compartment negative sampling was used, i.e., pairing proteins that share the same subcellular localizations, making the discrimination task harder. To create the training and test data for hard negative test, following pipeline was used. All unique protein sequences across each dataset were extracted and converted to FASTA format. Sequences exceeding 6,000 amino acids - the maximum accepted by the prediction server DeepLoc-2.0^41^ were excluded from localization prediction; these represent a negligible fraction of the dataset. Second, subcellular localization for all remaining proteins was predicted using DeepLoc-2.0, a deep learning model trained on experimentally verified UniProt/SwissProt annotations that predicts localization directly from raw amino acid sequence. Third, proteins sharing at least one predicted subcellular compartment and absent from the known positive set were paired as same-compartment hard negative candidates. Known positive pairs were explicitly excluded to prevent contradictory labelling. Hard negatives were sampled to match the positive set size, maintaining class balance, and a stratified train-test split was applied with zero cross-split pair leakage confirmed. The results are as follows:

**Table 4:** Results of hard negative experiment (same-compartment proteins).

Datasets	Accuracy (%)	AUROC (%)
*Human*	91.18 (0.09)	94.88(0.47)
*E. coli*	75.12 (0.29)	80.03(0.54)
*Drosophila*	82.51(0.67)	87.83(.06)
*C. elegans*	87.24(0.44)	89.37(0.20)

This table shows the evaluation results of hard-negative test, i.e., the negative protein pairs are chosen from same compartments

To our knowledge, this is the first reported hard-negative evaluation on Pan’s human benchmark and the Guo et al. multi-species datasets, establishing a new reference point for future comparisons on these widely adopted benchmarks. As shown in the updated Table 4, the model maintains high predictive performance in hard negative test, particularly in *Human* data (91.18% accuracy) and *C. elegans* (87.24%). A predictable decrease in accuracy for *E. coli* (75.12%) is likely due to the high molecular crowding and shared physiological environment inherent to prokaryotic cells. However, the overall stability of these scores confirms that the NSGA-II optimized Random Forest is effectively utilizing the deep biophysical signals captured by the ProtT5-XL embeddings.

### Ablation study

To unambiguously confirm the independent and additive contribution of both UMAP and NSGA-II in the proposed study, an ablation study has been performed comparing performance with no dimensionality reduction, PCA-based reduction, an autoencoder-based reduction and employing UMAP with default random forest parameters without using NSGA-II.

For choosing the number of dimensions in PCA, the elbow plot of the cumulative variance was drawn on *Human* data. While 90% of total variance is retained at 341 components, the elbow of the explained variance curve occurs at approximately 100 components- beyond which additional components contribute marginally. For this reason and to reduce computational complexity, 100 components were adopted as the PCA dimensionality across all datasets. For the autoencoder, a symmetric encoder-decoder architecture was implemented, trained with MSE reconstruction loss using the Adam optimizer over 100 epochs. A latent dimensionality of 100 was used to ensure a fair and directly comparable basis across all three reduction methods. The corresponding results are reported in the revised Table 5 below.

**Table 5:** Ablation study accuracies for different datasets (all in %).

Datasets	With NSGA-II framework			Without NSGA-II	Only NSGA-II
	With UMAP reduction (present study)	With PCA reduction	With autoencoder reduction	Framework using only UMAP	Framework on Original ProtT5 embeddings
*Human*	99.81 ± 0.04	92.11 ± 0.09	93.39 ± 0.11	98.00 ± 0.02	96.99 ± 0.01
*E. coli*	99.99 ± 0.01	95.22 ±0.30	95.89 ± 0.04	99.10 ± 0.01	97.11 ± 0.03
*Drosophila*	99.32 ±0.20	89.73 ± 0.12	91.20 ± 0.12	96.43 ± 0.02	94.58 ± 0.80
*C. elegans*	99.99 ± 0.01	94.89 ± 0.35	96.88 ± 0.23	98.89 ± 0.01	97.56 ± 0.43

This table presents a detailed ablation study including employment of PCA, autoencoder, and UMAP under NSGA-II-RF framework, employing UMAP directly with default random forest parameters and employing NSGA-II directly on Prott5 embeddings

Replacing UMAP with PCA produces the largest performance degradation across all datasets, confirming that linear projection fails to capture the nonlinear manifold structure of the protein interaction feature space. The autoencoder, although nonlinear, also underperforms, confirming that reconstruction-optimized representations are inferior to topology-preserving representations for this classification task. Removing NSGA-II and substituting default random forest parameters while retaining UMAP produces a further independent performance reduction, confirming that the Pareto-optimal hyperparameter configurations identified by multi-objective optimization are genuinely superior to unoptimized defaults. While the absolute accuracy gaps may appear modest at near-ceiling performance levels, at this accuracy level, even the slightest absolute improvement corresponds to a reduction in misclassified pairs in a proteome-wide screening context.

### Coverage analysis

**Fig. 5:**
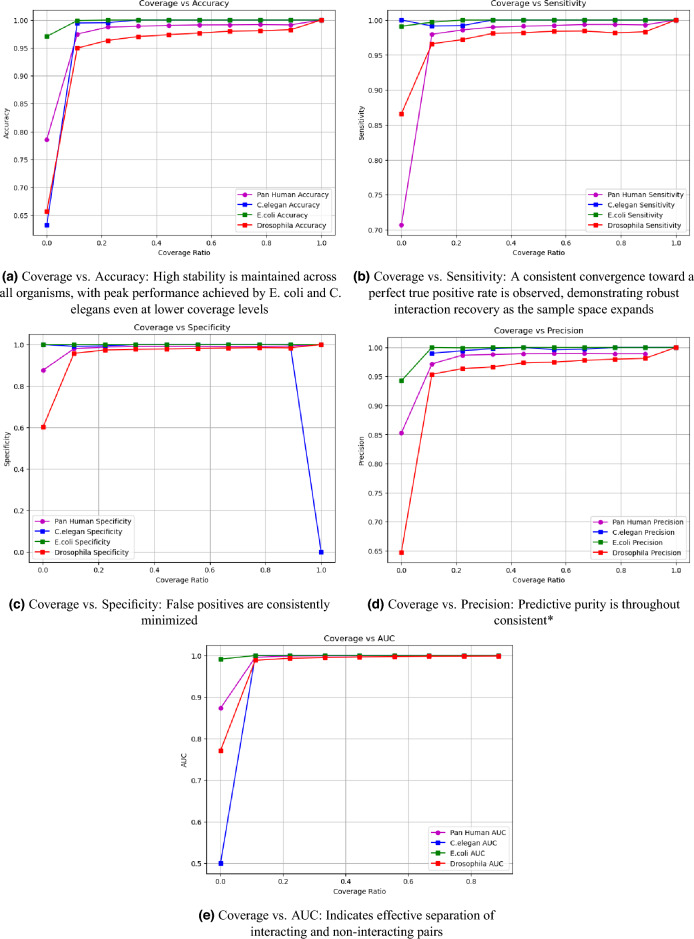
Association between coverage ratio and the five metrics accuracy, sensitivity, specificity, precision and AUC. Note*: Data points at a coverage ratio of 0 are omitted for metrics where the denominator is zero, as these values are mathematically undefined. This shows the association between coverage ratio and different metrics. (**a**) depicts coverage vs. accuracy which shows how the accuracies change with coverage going from 0 to 100%. (**b**), (**c**), (**d**) and (**e**) shows the similar analysis but for different metrics i.e., sensitivity, specificity, precision and AUC respectively.

The robustness of the proposed model has been examined for highly sparse datasets, considering the limited availability of existing PPIs. The “Coverage” is defined as the proportion of training samples to the total number of samples^24^. It basically tests a model’s ability to generalize for unseen or sparse data and can determine the computational resources needed for analysis. Lower coverage results in a sparser training set. Figure 5 exhibits the impact of varying coverages on various metrics such as accuracy, sensitivity, specificity, precision and AUC. While metrics for all datasets show an upward trend as coverage increases, the accuracy for *Human* data remains impressively high at around 98%, above 95% for *Drosophila* and nearly 100% for both C.elegan and E.Coli, even with just 0.2 coverage. At 0.4 coverage, for each data, sensitivity, specificity, and precision, each achieves results of at least above 98% and the AUC is nearly 99.95%, demonstrating consistent robustness and validity of the proposed model. These results further exhibit that the model remains effective even with sparse datasets and can be reliably applied to real-world predictions for other species.

### Explainability

**Fig. 6:**
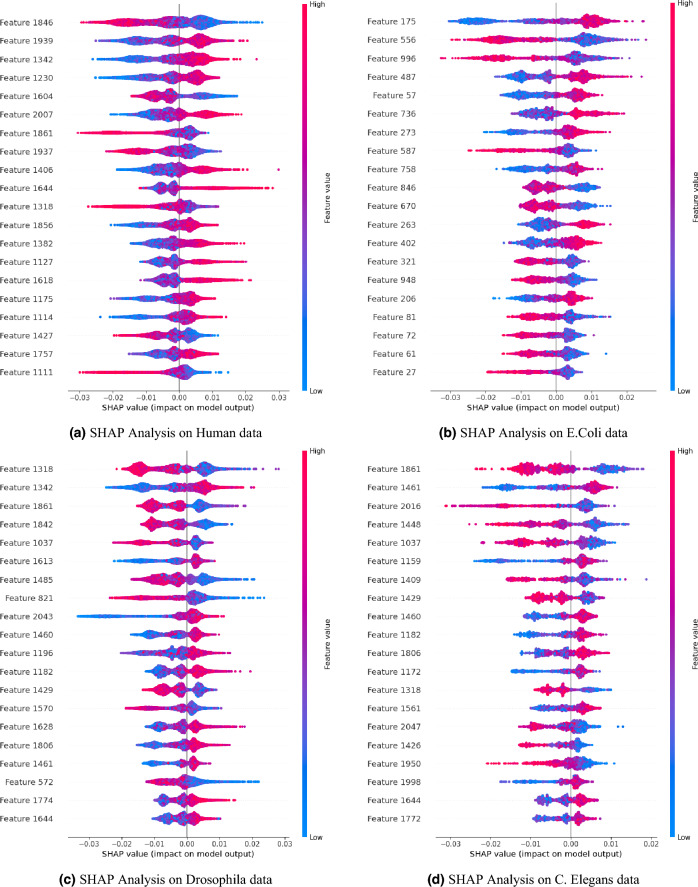
SHAP summary plots of proposed model on various organisms. This shows the SHAP summary plots of proposed model on various organisms. Each figure shows the features that have maximum contribution in this predictive model and how did they contribute. *Human* (**a**), *Drosophila* (**c**) and *C. elegans* (**d**) shows impact of the second protein is much higher while *E. Coli* (**b**) shows impact of the first protein the prediction purpose.

Recent advances in eXplainable AI (XAI) have improved the interpretability of machine learning models by enabling analysis of how input features contribute to predictions^33^. In this study, SHAP (SHapley Additive exPlanations)^34^ values were computed using the TreeExplainer on the optimized Random Forest classifier. SHAP analysis was performed on the original 2048-dimensional representation to examine feature contributions in the full input space, thereby enabling a more detailed assessment of how the original embedding dimensions influence the model’s predictions. The results are visualized using SHAP summary plots (Fig.  6), which display both the average feature importance for each dataset and the distribution of effects across all samples.

Across all datasets, it was observed that only a small subset of embedding dimensions, out of the 2048 total features, exerted substantial influence on classification outcomes. Since embeddings of each pair of concatenating / non-concatening protein is a 2048-dimensional representation (1024 per protein), features *\ge 1025* belong to the second protein and features *łe 1024* belong to the first one. Notably, there are variations in the relative contributions of the two protein partners across different organisms. For *Human* (Fig. 6), *C. elegans* (Fig. 6) and *Drosophila* (Fig. 6), the most influential features predominantly originated from the second protein embedding. On the contrary, in *C. elegans*, many features exhibited more mixed distributions. However, features from *Human* and *Drosophila* showed clearer monotonic patterns, while *C. elegans* showed more mixed distributions, indicating greater context dependency. For the *E. coli* dataset (Fig. 6) the most influential features originated primarily from the first protein embedding with narrower spread of SHAP values, indicating that individual feature contributions were weaker and more symmetric compared to others.

To assess the relative contributions of the two proteins in the concatenated embedding, the mean absolute SHAP values for the first 1024 dimensions (Protein A) and the last 1024 dimensions (Protein B) were aggregated. The corresponding numerical results are summarized in the following Table 6.

**Table 6:** Mean SHAP contribution of protein pairs in interaction prediction.

Dataset	Protein A	Protein B
*Human*	0.000118 (16.99%)	0.000575 (83.01%)
*E. coli*	0.000603 (89.43%)	0.000071 (10.57%)
*Drosophila*	0.000130 (19.16%)	0.000550 (80.84%)
*C. elegans*	0.000067 (10.78%)	0.000557 (89.22%)

This table depicts the percentage of contribution each protein in a protein pair makes to predict the interaction. This analysis has been done using SHAP

In *Human*, *Drosophila*, and *C. elegans*, the second protein embedding (Protein B) accounted for the vast majority of predictive influence (80–89%), whereas the first protein (Protein A) contributed only marginally. Conversely, in *E. coli*, the trend was reversed, with Protein A contributing nearly 90% of the predictive signal. The consistency of the proposed model in detecting the feature asymmetries shows that the model adapts to organism-specific interaction mechanisms rather than giving arbitrary attributions, suggesting that it is flexible and generalizable across species.

To provide a concrete biological case study, a Spearman correlation analysis was performed between the top-contributing Prott5 dimensions (according to SHAP) and four important biophysical properties of the dominant interaction partner viz., hydrophobicity, isoelectric point, molecular weight and instability index over all four species using ProtParam^36^. The reported correlations are all statistically significant (p<0.01; for majority cases p *≈* 0). This confirms the previous claim of the dominant dimensions encoding genuine, organism-specific biological signals rather than coincidental positional artifacts. Results for top 10 dimensions across all four species are given in following Tables 7,  8,  9,  10.

**Table 7:** *Human* dataset (Protein B dominant- SHAP contribution: 83.01%). Spearman correlation between top SHAP features and biophysical properties. All correlations marked * are significant at *p < 0.01*.

SHAP feature	Hydrophobicity (R)	Isoelectric point (R)	Molecular Weight (R)	Instability (R)	Biological signature
1846	+ 0.348*	− 0.012*	− 0.354*	− 0.388*	Compact, stable, hydrophobic - ordered hub
1342	− 0.401*	− 0.106*	+ 0.166*	+ 0.435*	Large, flexible, hydrophilic - disordered hub
1939	− 0.092*	− 0.145*	+ 0.255*	+ 0.164*	Large, acidic- transcriptional regulator
1230	− 0.456*	− 0.122*	+ 0.177*	+ 0.178*	Hydrophilic- surface-exposed partner
1604	+ 0.080*	− 0.087*	− 0.104*	− 0.146*	Compact, mildly stable
2007	− 0.021*	+ 0.097*	+ 0.218*	+ 0.018*	Large, basic- nuclear/regulatory
1861	+ 0.354*	− 0.019*	− 0.236*	− 0.523*	Ordered globular hub- strongest stability signal
1937	+ 0.263*	+ 0.034*	− 0.239*	− 0.409*	Hydrophobic, stable- interface-mediated
1406	+ 0.060*	+ 0.025*	− 0.066*	− 0.132*	Compact, mildly stable
1644	− 0.072*	+ 0.095*	+ 0.248*	+ 0.328*	Large, flexible- disordered partner

Strongest stability signal: Feature 1861 vs Instability (*R = -0.523*, *p ≈ 0*)

Shows Spearman correlation analysis between top 10 SHAP features and biophysical properties on *Human* data

**Table 8:** *Drosophila* Dataset (Protein B dominant- SHAP contribution: 80.84%). Spearman correlation between top SHAP features and biophysical properties. All correlations marked * are significant at *p < 0.01*.

SHAP feature	Hydrophobicity (R)	Isoelectric point (R)	Molecular weight (R)	Instability (R)	Biological signature
1318	+ 0.597*	− 0.110*	− 0.114*	− 0.651*	Compact, highly stable- strongest signal in *Drosophila*
1342	− 0.441*	− 0.040*	+ 0.093*	+ 0.555*	Large, flexible, hydrophilic- disordered hub
1861	+ 0.427*	− 0.086*	− 0.200*	− 0.569*	Compact, hydrophobic- ordered globular hub
1842	+ 0.363*	− 0.011*	− 0.257*	− 0.513*	Compact, stable- ordered interaction partner
1037	+ 0.391*	− 0.122*	− 0.138*	− 0.491*	Hydrophobic, stable- interface-mediated
1613	− 0.281*	− 0.027*	+ 0.307*	+ 0.530*	Large, flexible- disordered hub
1485	+ 0.352*	+ 0.133*	− 0.144*	− 0.550*	Stable, mildly basic- ordered regulatory
821	+ 0.089*	− 0.003	+ 0.007	− 0.079*	Weakly biophysical- likely evolutionary signal
2043	− 0.289*	− 0.022*	+ 0.325*	+ 0.501*	Large, flexible- disordered partner
1460	− 0.333*	+ 0.042*	+ 0.174*	+ 0.512*	Flexible, hydrophilic- surface-exposed partner

Strongest stability signal: Feature 1318 vs Instability (*R = -0.651*, *p ≈ 0*). Feature 1861 cross-species consistent with *Human* (*R = -0.569*)

Shows Spearman correlation analysis between top 10 SHAP features and biophysical properties on *Drosophila* data

**Table 9:** *C. elegans* Dataset (Protein B dominant — SHAP contribution: 89.22%). Spearman correlation between top SHAP features and biophysical properties. All correlations marked * are significant at *p < 0.01*.

SHAP feature	Hydrophobicity (R)	Isoelectric point (R)	Molecular weight (R)	Instability (R)	Biological Signature
1861	+ 0.498*	− 0.070*	− 0.264*	− 0.599*	Compact, hydrophobic- ordered hub (cross-species consistent)
1461	− 0.333*	− 0.111*	+ 0.379*	+ 0.541*	Large, flexible- disordered hub
2016	+ 0.093*	+ 0.190*	− 0.224*	− 0.316*	Compact, mildly stable
1448	+ 0.189*	+ 0.096*	− 0.303*	− 0.456*	Stable, compact- ordered partner
1037	+ 0.424*	− 0.001	− 0.137*	− 0.547*	Hydrophobic, stable- ordered interface (cross-species consistent)
1159	− 0.386*	− 0.089*	+ 0.426*	+ 0.502*	Large, flexible- disordered hub
1409	+ 0.294*	+ 0.057*	− 0.077*	− 0.346*	Hydrophobic, stable
1429	+ 0.500*	− 0.003	− 0.196*	− 0.496*	Compact, hydrophobic- ordered hub
1460	− 0.348*	− 0.090*	+ 0.249*	+ 0.485*	Flexible, hydrophilic- surface-exposed partner
1182	− 0.396*	− 0.089*	+ 0.306*	+ 0.513*	Large, flexible- disordered partner

Feature 1861 cross-species consistent across all three eukaryotic species (*R = -0.599*). Feature 1037 consistent with *Drosophila* (*R = -0.547*)

Shows Spearman correlation analysis between top 10 SHAP features and biophysical properties on *C. elegans* data

**Table 10:** *E. coli* Dataset (Protein A dominant — SHAP contribution: 89.43%). Spearman correlation between top SHAP features and biophysical properties. All correlations marked * are significant at *p < 0.01*.

SHAP feature	Hydrophobicity (R)	Isoelectric point (R)	Molecular weight (R)	Instability (R)	Biological signature
175	− 0.488*	− 0.011	− 0.299*	− 0.056*	Hydrophilic, compact- surface interaction partner
556	+ 0.330*	− 0.033*	+ 0.303*	+ 0.159*	Hydrophobic, larger- multi-subunit component
996	+ 0.386*	+ 0.006	+ 0.405*	+ 0.020	Large, hydrophobic- complex component
487	− 0.552*	− 0.165*	− 0.198*	+ 0.047*	Most hydrophilic- aqueous surface contact
57	− 0.179*	+ 0.022*	− 0.268*	− 0.262*	Compact, stable- strongest stability in *E. coli*
736	− 0.340*	+ 0.038*	− 0.140*	− 0.051*	Hydrophilic- surface-exposed
273	− 0.300*	− 0.207*	− 0.093*	− 0.118*	Acidic, compact- signal transduction
587	+ 0.510*	+ 0.013	+ 0.337*	+ 0.045*	Hydrophobic, larger- membrane/interface
758	− 0.354*	+ 0.044*	− 0.466*	− 0.088*	Compact, hydrophilic- small regulatory
846	+ 0.207*	− 0.212*	+ 0.248*	+ 0.275*	Flexible, acidic- regulatory protein

Dominant axis is hydrophobicity (not instability), reflecting the compact and metabolically specialized prokaryotic interactome. Strongest signal: Feature 487 vs Hydrophobicity (*R = -0.552*)

Shows Spearman correlation analysis between top 10 SHAP features and biophysical properties on *E. coli* data

Across all four species, three interpretable axes emerge consistently: structural order/disorder, hydrophobic interface and protein size. The first axis dominates through all three eukaryotic datasets, encoding stable ordered and intrinsically ordered proteins, and ranking among top SHAP dimensions consistently, with individual correlations of R = -0.523 (Feature 1861, *Human*), R = -0.651 (Feature 1318, *Drosophila*), and R = -0.599 (Feature 1861, *C. elegans*). Moreover, feature 1861 appears in eukaryotic datasets with almost identical instability correlations (R = -0.523 (*Human*), R = -0.569 (*Drosophila*), and R = -0.599 (*C. elegans*)), indicating that this dimension encodes the ordered hub protein property across eukaryotic organisms. This cross-species reproducibility proves that the model learns biophysical interactions. In case of E.Coli, the dominant axis is hydrophobicity while instability correlations are uniformly weak (R = -0.262). This points towards the fundamental architecture difference of the prokaryotic interactome, showing that the proteins are more stable and compact with the interactions primarily being governed by hydrophobicity patterns unlike the eukaryotes. The absence of the instability axis in E.Coli and its strong presence in all three eukaryotic species provides a molecular-level explanation for the species-specific SHAP asymmetry reversal observed in our study.

## Significance and complexity

### Statistical significance

To determine the statistical significance of the proposed method, the confusion matrices for the four datasets are obtained from the experiment pipeline and the Chi-squared test is conducted. Chi-squared test is a non-parametric test. It used to evaluate the significance of the dissimilarity between the observed values and the expected values. Using values from the confusion matrices, the respective *p*-values for the calculated Chi-Squared distributions with 1 degree of freedom are computed to be 0.007, 0.0, 0.0 and 0.0. Given that the *p*-value in each case is less than the significance level of 0.05, it is implied that proposed model’s predictions are significantly better than random chance, showcasing its ability to differentiate between the classes effectively.

### Time complexity

The algorithm of the presented PPI prediction technique consists of multiple computational steps, each with a distinct time complexity. The primary steps and their associated complexities are given in detail below:

*Embedding protein sequences:* Employment of ProtT5-XL-UniRef50 is computationally intensive due to the self-attention mechanism of T5 architecture, which has a time complexity of $O(n^2\cdot d)$ where, *n* is the input sequence length and *d* is the embedding dimension. Each transformer layer includes a position-wise feed-forward network contributing $O(n\cdot d^2)$. Since there are 24 transformer layers and *d*=1024, the overall complexity becomes $O(24\cdot N(n^2\cdot d+ n\cdot d^2))$, where, *N* is the total number of data points.

*Dimensionality reduction:* The overall time complexity of UMAP can be summarized as $O(N\cdot log(N)\cdot d+N\cdot k\cdot t)$, where, k is number of neighbors and t is number of optimization iterations. The first and second term in this formation are attributed by construction of graph and optimization respectively.

*Parameter optimization using NSGA-II:* NSGA-II has a overall time complexity of *O*($P^2$) per generation, which is governed by the nondominated sorting part of the algorithm, *P* indicating the population size. The time complexity for training a Random Forest is approximately $O(T\cdot m\cdot log(m))$, where, *T* is the number of trees and *m* is the number of data points to train a tree. If *g* is the number of generations in the the NSGA-II algorithm, the overall complexity for this step becomes $O(g\cdot (P\cdot T\cdot m\cdot log(m)+ P^2))$

Combining the above mentioned complexities from each step, the overall time complexity of the proposed workflow can be approximated as:

$$O(24\cdot N(n^2\cdot d+n\cdot d^2))+O(N\cdot log(N)\cdot d+N\cdot k\cdot t)+O(g\cdot (P\cdot T\cdot m\cdot log(m)+ P^2))$$

Given that embedding and parameter optimization are the most computationally intensive steps, the above term can be compiled as:

$$O(24\cdot N(n^2\cdot d+n\cdot d^2))+O(g\cdot (P\cdot T\cdot m\cdot log(m)+ P^2))$$

*≈* $O(N\cdot n^2+ g\cdot (P\cdot T\cdot m\cdot log(m)+ P^2))$, since the embedding part is dominated by self-attention mechanism part and *d* is a constant term.

*Resource consumption and actual runtime:* The experiments were conducted on a high-performance workstation running CentOS Linux 7, equipped with dual Intel(R) Xeon(R) Gold 6242 CPUs (32 total physical cores @ 2.80GHz) and 187GB of RAM. The software environment comprised Python 3.x, scikit-learn 1.5.2, pymoo 0.6.1.3, numpy 2.0.1, pandas 2.2.2, PyTorch 2.4.1, and mpi4py 3.1.4. For the largest dataset, which is the Pan *Human* data, obtaining prott5 embedding took around 5 minutes, with UMAP reduction taking few seconds and the NSGA-RF optimization stage took around 30 minutes for 50 generations. The MPI parallelization distributed individual fitness evaluations across all available processes simultaneously, with rank 0 handling data I/O and result aggregation while worker ranks performed independent RF training and evaluation.

## Comparisons

### Comparison with state-of-the-art methods

To evaluate the effectiveness of proposed approach, the obtained results were compared against several widely used machine learning and deep learning baselines reported in the literature. Amongst these are recent benchmark works ^18–24,40^ based on graph-based approaches, stacked auto-encoders, language model based frameworks etc. Table 11 summarizes and presents an overview of the performance comparison in *Human* data. Table 12, table  13, and table 14 represent performance comparisons of the proposed model with some of the current reference techniques on the *E. coli*, *Drosophila*, and *C. elegans* dataset, respectively.

**Table 11:** Performance comparison for *Human* data.

Article	Acc.(%)	**Sens.(%)**	Spec.(%)	MCC(%)	f1(%)	AUROC(%)
^18^	94.93±0.1	95.50 ± 0.20	–	–	94.90 ± 0.10	94.90 ± 0.10
^40^	96.82	–	–	–	–	–
^19^	97.77	98.06	98.06	–	–	–
^23^	97.90	98.50 ± 0.90	97.30 ± 0.15	–	97.90	97.90
^22^	98.13	98.84	96.18	95.20	98.73	98.28
^21^	98.26 ± 0.02	97.40 ± 0.00	97.90 ± 0.03	96.40 ± 0.03	98 ± 0.02	–
^20^	99.02 ± 0.13	99.10 ± 0.90	98.50 ± 1.10	98.00 ± 0.30	99.00 ± 0.10	–
^24^	99.10 ± 0.10	99.41 ± 0.10	98.90 ± 0.10	–	99.10 ± 0.10	–
Present study	**99.81** ± **0.04**	**99.86** ± **0.03**	**99.90** ± **0.07**	**99.32** ± **0.09**	**99.92** ± **0.01**	**99.90** ± **0.00**

Shows comparison of various metrics calculated on *Human* data using the proposed model with other state-of-the-art models. The same comparison is shown for *E. coli*, *Drosophila* and *C. elegans* in Tables 12, 13 and, 14 respectively

**Table 12:** Performance comparison for *E. coli* data.

Article	Acc.(%)	Sens.(%)	Spec.(%)	MCC(%)	f1(%)	AUROC(%)
^40^	96.05	96.89	95.28	–	–	–
^21^	95.20	–	–	90.90	95.20	98.30
^20^	99.74	99.62	99.82	99.46	99.68	–
^24^	98.92 ± 0.37	98.42 ± 0.34	99.42 ± 0.73	–	98.92 ± 0.54	–
This study	**99.99** ± **0.01**	**99.98** ± **0.0**	**1.00** ± **0.00**	**99.97** ± **0.0**	**99.99** ± **0.00**	**1.00** ± **0.00**

**Table 13:** Performance comparison for *Drosophila* data.

Article	Acc.(%)	Sens.(%)	Spec.(%)	MCC(%)	f1(%)	AUROC(%)
^40^	97.23	99.35	95.28	–	–	–
^21^	96.60	–	–	95.60	97.80	99.60
^20^	**99.99**	**100**	**100**	**99.99**	**99.99**	–
^24^	99.80 ± 0.01	99.61 ± 0.17	100 ± 0.02	–	99.80 ± 0.15	–
This study	99.32 ± 0.20	99.79 ± 0.01	99.77 ± 0.01	99.83 ± 0.01	99.97 ± 0.00	99.98 ± 0.01

**Table 14:** Performance comparison for *C. elegans* data.

Article	**Acc.(%)**	Sens.(%)	Spec.(%)	MCC(%)	f1(%)	AUROC(%)
^40^	97.23	99.35	95.28	–	–	–
^21^	99.00	–	–	97.90	99.00	99.7
^20^	99.44	99.83	98.78	98.80	99.56	–
^24^	99.26 ± 0.23	99.16 ± 0.38	99.35 ± 0.28	–	99.25 ± 0.33	–
This Study	**99.99 ± 0.01**	**1.00 ± 0.00**	**99.94 ± 0.04**	**99.95 ± 0.04**	**99.98 ± 0.00**	**1.00 ± 0.00**

The comparative analysis of PPI prediction methods reveals the exceptional performance of the proposed approach. The proposed model outperforms all compared models by significant margins across all datasets in multiple key metrics. Although the model proposed by^20^ slightly exceeded the proosed model on the *Drosophila* dataset, the difference was marginal and does not undermine the overall reliability of proposed approach. The highest scores in each metric are marked in bold letters. For *Human* data, the high F1 score of 99.2%, MCC score of around 98.32% and area under ROC curve of 99.9% indicates robust predictive capability of the suggested technique across different performance metrics. This validates the model’s efficacy in enhancing PPI prediction accuracy through the integration of NSGA-II optimization and Random Forest classification. It also underscores the importance of incorporating protein language model in boosting the predictive capability of the model.

### Comparison with XGBoost

To further validate proposed methodology, Random Forest is replaced with XGBoost; then the same pipline is followed with the same optimization procedure. The obtained results confirm the superior stability and generalization capability of Random Forest in this setting. Table 15 presents the performance of the optimized XGBoost model, while the corresponding optimized hyperparameters are detailed in Table 16.

**Table 15:** Performance of optimized XGBoost model on the datasets.

Dataset	Acc.(%)	Sens.(%)	Spec.(%)	MCC(%)	f1(%)	AUROC(%)
*Human*	99.76 ± 0.01	99.75 ± 0.02	99.86 ± 0.20	99.32 ± 0.30	99.90 ± 0.10	99.90 ± 0.00
*E. coli*	99.96 ± 0.001	99.99 ± 0.01	99.97 ± 0.00	99.98 ± 0.00	99.99 ± 0.00	99.99 ± 0.00
*Drosophila*	95.73 ± 0.06	96.30 ± .04	95.77 ± 0.01	95.96 ± 0.02	96.13 ± 0.00	94.38 ± 0.01
*C. elegans*	99.95 ± 0.01	1.00 ± 0.00	99.99 ± 0.00	99.96 ± 0.04	99.98 ± 0.00	1.00 ± 0.00

Shows the computed metric values when in the proposed pipeline Random Forest is replaced with XGBoost

**Table 16:** Optimized parameters for XGBoost.

Dataset	n_estimators	max_depth	learning_rate	min_child_weight	subsample	colsample_bytree
*Human*	544	16	0.1806	7	0.9578	0.9589
*E. coli*	82	1	0.8026	1	0.9977	0.7394
*Drosophila*	417	8	0.1611	2	0.9662	0.9261
*C. elegans*	25	1	0.8030	4	0.9300	0.6953

Table 16 denotes the optimized hyperparameters in this case

Both optimized random forest (RF) and XGBoost achieved near-perfect performance on the *E. coli* and *C. elegans* datasets. On the *Human* dataset, XGBoost marginally outperformed RF in accuracy, while RF achieved a higher MCC, indicating more balanced classification. In contrast, for *Drosophila*, RF maintained strong performance (99.32% accuracy, 98.83% MCC, 99.9% AUROC), whereas XGBoost showed a marked decline (95.73% accuracy, 95.96% MCC, 94.38% AUROC), suggesting weaker generalization.

Overall, RF demonstrated more consistent performance across all organisms, with lower variance and greater robustness to high-dimensional embedding noise. Although XGBoost performed competitively on some datasets, its performance degraded notably on *Drosophila*, indicating higher sensitivity to redundancy or noise. These results suggest that RF is better suited for protein–protein interaction prediction using high-dimensional protein embeddings.

## Discussion

The results of the proposed model are demonstrated in Table 11 and it shows that this study surpasses the recent cutting-edge techniques with even deep learning techniques and has reduced runtime complexity and requires lesser memory. Since the PPI prediction problem that largely depends on the dataset, the same set of model parameters might not universally perform well across different datasets. The proposed method addresses this critical limitation by leveraging the NSGA-II algorithm to dynamically optimize the Random Forest parameters for each specific dataset.

NSGA-II is known for being computationally expensive compared to commonly used Bayesian Optimization or Random Search; however, it holds the multi-objective nature that is necessary for this specific problem. Standard hyperparameter optimization methods such as random search^35^ and Bayesian optimization^39^ are designed for single-objective problems and would require collapsing our accuracy and diversity objectives into an arbitrary weighted scalar, thereby losing Pareto-optimal trade-off information. Furthermore, while Bayesian optimization is inherently sequential due to its posterior update mechanism, NSGA-II’s population-based structure is naturally parallelizable.

Even though there are many other multi-objective evolutionary algorithm available, choosing NSGA-II over them has some key reasons: (1) Due to employment of crowding distance, NSGA-II often finds a more uniform distribution of solutions along the Pareto front, (2) Its built-in elitism mechanism through its non-dominated sorting and crowding distance sorting proves to be better than other algorithm’s archiving strategy, (3) constraints are handled more naturally through its tournament selection process, (4) It typically requires fewer parameters to be tuned compared to other evolutionary algorithms. In this study, both accuracy and diversity were considered as dual objectives to construct a vigorous and fruitful prediction model. While diversity is essential for enhancing the generalizability of the ensemble and mitigating overfitting, accuracy directly reflects the model’s predictive performance, which is often the most critical requirement in real-world applications. The Pareto-optimal front provides a set of non-dominated solutions, representing the trade-off between these two objectives. To select the final solution, the populations from the pareto front with ’maximizing accuracy’ is prioritized. Although diversity was not the primary criterion for selection, its consideration during optimization ensures that the chosen solution maintains acceptable levels of diversity, as it lies within the Pareto-optimal set. This approach balances the importance of both objectives while aligning with the specific demands of the application, where accurate predictions are paramount.

Moreover, while trying to explain the model mechanism, the striking differences in mean SHAP contributions (Table 6) suggest that while the model adapts its reliance on different embedding halves depending on the organism, the learned decision boundaries are strongly asymmetric. The prott5 embeddings encode protein sequences along high structured and correlated axes. The 1024 dimensions are not independent information channels. The SHAP analysis confirms that only a small subset of the total 2048 dimensions exerts substantial influence on the final classification which are three interpretable biological axes: structural order/ disorder, hydrophobicity and protein size. The low intrinsic dimensionality of the protein interaction is consistent with this finding. Further investigation, possibly through order-invariant encodings(e.g., elementwise addition, subtraction, or max-pooling of embeddings), may discover more about biological interpretations.

In a scenario where a new protein sequence from a specific species arises, it will be embedded through the Prott5 model, then UMAP will be re-run on the whole feature matrix to capture the new protein’s geometric architecture into the global manifold structure. Following this, the interaction status of the new protein’s candidate pairs can be classified using the previously optimized random forest hyperparameters. However, for large-scale deployment scenarios where proteins are added in large batches, the complete pipeline would be re-executed on the updated proteome to identify a new set of Pareto-optimal random forest hyperparameters calibrated to the updated embedding geometry.

## Conclusion

In this research, the creation of an unconventional model, aimed to predict PPIs, exclusively from protein sequences has been explored. It is founded on the belief that protein sequences inherently possess adequate information for PPI prediction, a notion that is gaining widespread acceptance in recent scientific studies. The primary objective is to enhance the performance of the random forest algorithm by creating diverse and effective training sets, which in turn produce accurate and varied classifiers. Since there is a trade-off between diversity and accuracy, these two criteria are optimized simultaneously to generate precise and diverse decision trees, and to identify the (near) optimal number of trees. The novelty of the work lies in the development of the hybrid of a multiobjective genetic algorithm and random forest algorithm while deriving amino acid embeddings from protein language model Prot-t5xl-uniref50. This PLM provides scalable, fast, and accurate representations for large-scale proteome studies. The embeddings are cocatenated to understand the mutual effect of interacting or no-interacting proteins. UMAP is used here for reducing the high dimensionality of the data while preserving its topological structure. The hyperparameters of the random forest algorithm are tuned by training on the training dataset and then validated over the testing dataset.

Unlike traditional grid or random search methods, NSGA-II uses advanced evolutionary strategies to explore the hyperparameter space more intelligently. This adaptive parameter optimization approach ensures that the model can effectively adjust its internal configurations to match the unique characteristics of different PPI datasets. This enhances the model’s robustness and adaptability, thereby overcoming the traditional challenge of fixed parameter settings that may perform suboptimally across diverse biological data contexts. The approach demonstrates a generalizable framework that can be extended to other machine learning models beyond Random Forest, potentially offering a universal strategy for adaptive model parameter optimization in biological prediction tasks.

## Data Availability

All data generated or analysed during this study are included in this published article
